# Positive Reinforcement Training in Captive Wild Mammals: An Integrative Review

**DOI:** 10.3390/ani16182914

**Published:** 2026-09-16

**Authors:** Lara Caveanha Gragnanello, Mateus José Rodrigues Paranhos da Costa, Cristiane Schilbach Pizzutto, Cynthia Fernandes Cipreste, Robert John Young, Aline Cristina Sant’Anna

**Affiliations:** 1 Graduate Program in Animal Science, Faculty of Agricultural and Veterinary Sciences, UNESP, Jaboticabal 14884-900, SP, Brazil; 2 Grupo de Estudos e Pesquisas em Etologia e Ecologia Animal (ETCO), Faculty of Agricultural and Veterinary Sciences, UNESP, Jaboticabal 14884-900, SP, Brazilaline.santanna@unesp.br (A.C.S.); 3 Department of Animal Science, Faculty of Agricultural and Veterinary Sciences, UNESP, Jaboticabal 14884-900, SP, Brazil; 4 Laboratory of Ecology and Evolution, Butantan Institute, São Paulo 05503-900, SP, Brazil; crissp@usp.br; 5 Belo Horizonte Zoo-Botanical Foundation, Belo Horizonte 30130-003, MG, Brazil; 6 School of Science, Engineering and Environment, University of Salford Manchester, Peel Building—Room G51, Salford M5 4WT, UK

**Keywords:** learning, animal welfare, operant conditioning, conservation, human care, reinforcement

## Abstract

**Simple Summary:**

This review evaluates the impact of positive reinforcement training (PRT) on wild mammals in ex situ environments, emphasizing its role in enhancing animal welfare. From the past decade, 46 studies were analyzed: 17 focused directly on PRT effects, while 29 explored its integration within broader management strategies. Guiding PRT with principles that promote animals’ well-being, safety, and effectiveness is important, requiring detailed session planning, specific techniques such as differential reinforcement, and systematic response monitoring. These practices ensure ethical, tailored, and efficient training, which is vital for daily management, veterinary procedures, and research on cognition. Training programs should be adaptable, considering behavioral and physiological traits like temperament, age, and social behavior to maximize success. Sharing detailed methodologies and employing innovations like AI-based video analysis can further improve practices. Collaboration between institutions—such as zoos and conservation centers—is key to disseminating best practices and supporting research. Despite around 6446 known mammal species being listed by the IUCN, only 46 studies cover about 0.54%, mainly focusing on primates, big cats, whales, and other similar groups relevant for management and conservation. These training initiatives aim to improve animal welfare, facilitate scientific research, and advance conservation efforts.

**Abstract:**

This review critically evaluates the impact of positive reinforcement training (PRT) on wild mammals maintained in ex situ environments, highlighting its role in enhancing animal welfare. The synthesis consolidates recent research on how PRT influences mammalian behavior and physiology with a focus on training techniques and types of reinforcers employed. A total of 46 studies from the past decade were analyzed: 17 directly examined the effects of PRT and 29 explored its application within broader ex situ management strategies that incorporated PRT alongside other approaches. Of these, 35 articles detailed the specific positive reinforcers used during training sessions. The findings demonstrate diversity and facilitate the adaptation to management routines, promote behavioral diversity, and decrease the occurrence of stress-related behaviors. Factors such as social interactions, individual motivation, and the variety of reinforcers significantly influence training outcomes. This review provides a foundational framework for applying operant conditioning with positive reinforcement to improve management protocols, veterinary procedures, and conservation efforts. It also emphasizes the need for further research to integrate PRT into projects involving biological sample collection and animal handling, with particular attention to individual animals’ needs and personality traits.

## 1. Introduction

Caring for wild mammals under human care, such as in zoos, conservation centers, and research centers, entails complex challenges that necessitate management strategies focused on animal welfare and the promotion of natural behaviors. Welfare refers to the animals’ physical and mental states, which are shaped by positive and negative experiences within their environment [1]. Ensuring that captive animals thrive requires creating conditions tailored to meet their basic needs, whilst also considering their affective states, which are expressed through their behaviors.

The concept of operant conditioning, first introduced by B.F. Skinner [2], can be used to improve captive animal welfare. This technique is based on the premise that behavior can be shaped by meaningful consequences that reinforce or punish specific actions [3]. Operant conditioning can be applied to optimize management procedures [4], monitor health [5] and foster more positive human–animal interactions [6].

Operant conditioning involves two primary types of consequences for the exhibited behavior: reinforcement and punishment [2]. Reinforcement is designed to increase the likelihood of repeated behavior. It can be positive, where a pleasant stimulus, such as food, is presented following the desired behavior [7], or negative, where an unpleasant stimulus is removed to encourage the behavior’s recurrence [8]. The sequence of behavior influenced by reinforcement or punishment can frequently be observed in both wild and captive animals. In a captive setting, positive reinforcement not only promotes behavioral consistency but also facilitates the development of trust between animals and trainers, contributing to improved overall well-being and fostering a safer, more engaging environment [8,9]. Conversely, punishment aims to decrease the probability of behavior occurring in the future. It can be positive, by applying an unpleasant stimulus in response to the behavior, or negative, by withholding a positive stimulus to reduce the likelihood of the behavior [10].

The stimulus–response–reinforcement contingency is a key component of operant conditioning, as it establishes the relationship between an animal’s behavior (response) and the resulting consequence (reinforcement or punishment). Additionally, the timing of reinforcement plays a significant role: it increases the likelihood of behavior repetition, contributing to improvements in management and the emotional well-being of animals [9]. It is also important to distinguish between primary reinforcers, which satisfy biological needs, and secondary reinforcers, which acquire value through associations with primary reinforcers. Primary reinforcers, such as food, directly satisfy needs and serve as potent motivators. In contrast, secondary reinforcers, like whistles or clickers, acquire value through association with primary reinforcers via classical conditioning [7]. These secondary reinforcers are particularly useful when primary reinforcers cannot be immediately provided, assisting animals in associating the expression of requested behaviors with positive outcomes [11].

Research indicates that positive reinforcement training (PRT) significantly improves the quality of life of mammals under human care, facilitating veterinary procedures [5,12,13,14] and routine management [12,15]. Additionally, this approach plays a role in conservation efforts [15,16,17,18] species preservation [18,19], and reproduction programs [20,21,22]. By fostering cooperative behavior between the animal and trainer, PRT simplifies routine interventions and underpins more effective management and health monitoring [23]. However, applying operant conditioning with positive reinforcement in wild mammals kept in captivity presents several challenges that must be addressed. One major difficulty lies in replicating training protocols accurately, as each animal responds uniquely to different approaches. Consequently, protocols cannot be rigid; they must be flexible and tailored to individual temperaments, reinforcement choices, and available resources and facilities. Furthermore, the current literature on PRT predominantly focuses on a limited number of species, often those with more complex management requirements necessitating safety considerations and species-specific adaptations.

This paper aims to investigate fundamental principles and strategies of training, emphasizing the importance of prioritizing positive reinforcement. Methodological limitations observed in current research will be addressed, highlighting the necessity for detailed descriptions to improve reproducibility and adherence to validated animal welfare guidelines. By synthesizing the training experiences and results of previous studies, this review strives to become a definitive resource that accelerates the widespread adoption of proven, effective techniques. Therefore, this review aims not only to describe the existing data but to synthesize, evaluate, and promote the dissemination of the most effective practices. By fostering a perspective, it seeks to contribute to the development of management programs that substantially enhance animal welfare.

## 2. Literature Search

**Figure 1:**
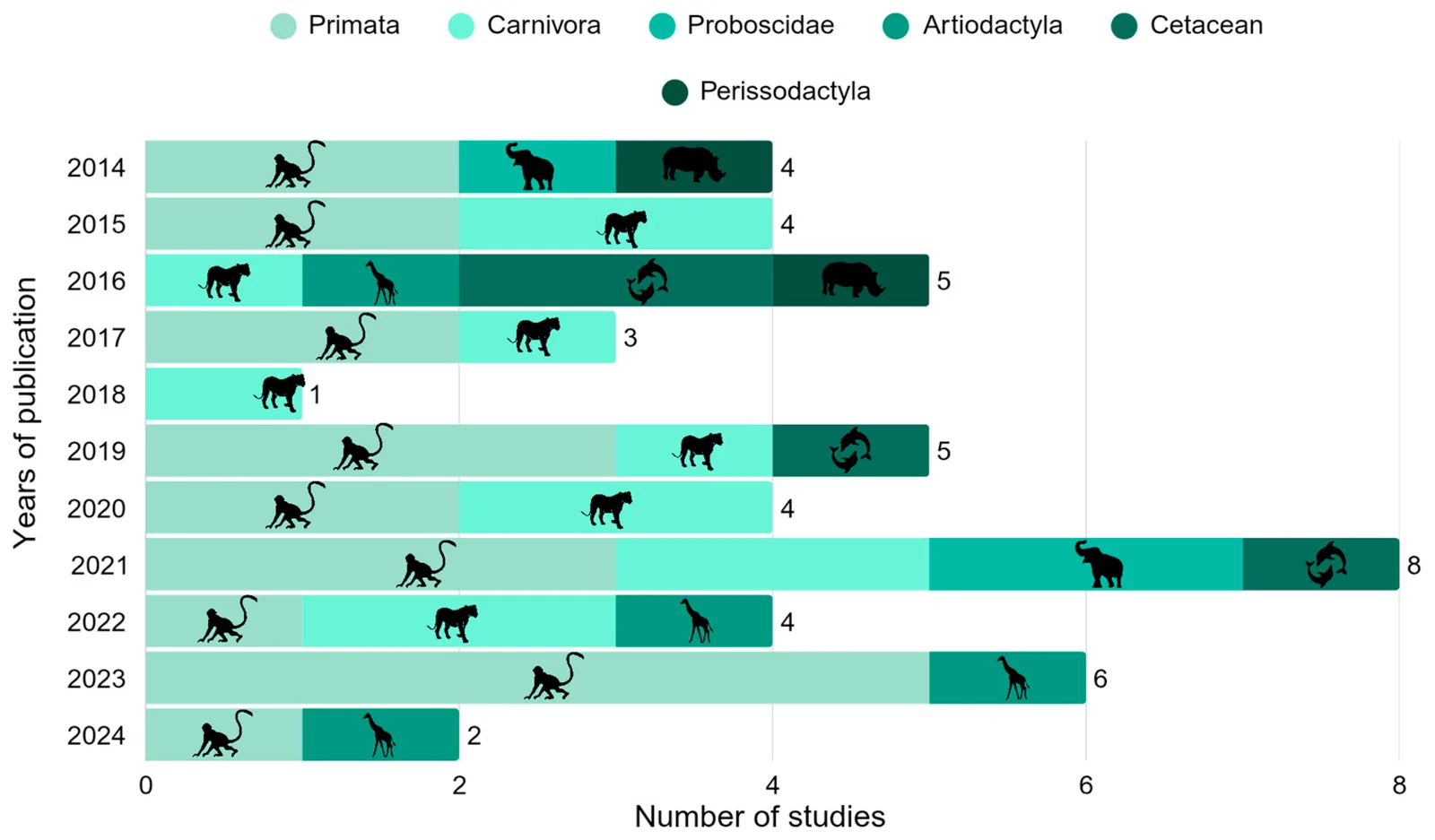
Number of publications in the scientific field of positive reinforcement training for animals, organized by year of publication. Animal icons help identify the respective orders. The absence of certain groups in specific years indicates a lack of publication related to those orders during those periods.

The literature was systematically searched across the PubMed, Scopus, and Web of Science databases. The keywords employed included “positive reinforcement training”, “positive reinforcement”, “animal training”, “training”, “operant conditioning”, “conditioning”, “captive”, “human care”, “wild”, “management”, “mammal” and “animal”. The inclusion criteria encompassed articles published in scientific journals in Portuguese, English, and Mandarin, covering the period from 2014 to August 2024 (Figure 1).

Studies focusing on the application of operant conditioning with positive reinforcement in wild non-human mammals, with an emphasis on conservation-related contexts, were included. Conversely, studies involving animals in vivariums, such as rhesus monkeys in laboratory settings (these animals are typically bred and managed under standardized protocols aimed at biomedical or fundamental behavioral research), as well as those with non-mammalian species (fish, reptiles, amphibians, echinoderms, and birds) and domestic mammals (cattle, horses, dogs, cats, pigs, and rats), were excluded.

During the literature review process, articles were initially screened based on their titles and abstracts to identify studies that focused on operant conditioning with positive reinforcement in wild mammals. The selected studies were then read in full to extract relevant data, focusing on key aspects such as the mammal species involved, sample size, specific training objectives—whether to improve management, modify behavior, or facilitate biological sample collection—as well as the training methodologies employed, and the primary reinforcement items utilized.

This review is organized into three sections. The first examines the impact of PRT on welfare and quality of life in ex situ mammals. The second reviews studies that have employed operant conditioning as a research protocol, including the validation of methodologies, veterinary management under experimental or routine conditions, and the use of cognitive or preference tests highlighting how PRT has been used as a methodological strategy to achieve specific scientific objectives. The third section explores the various types of primary and secondary reinforcers used in operant conditioning, providing an overview of the specific reinforcers implemented across the studies.

## 3. Results

**Figure 2:**
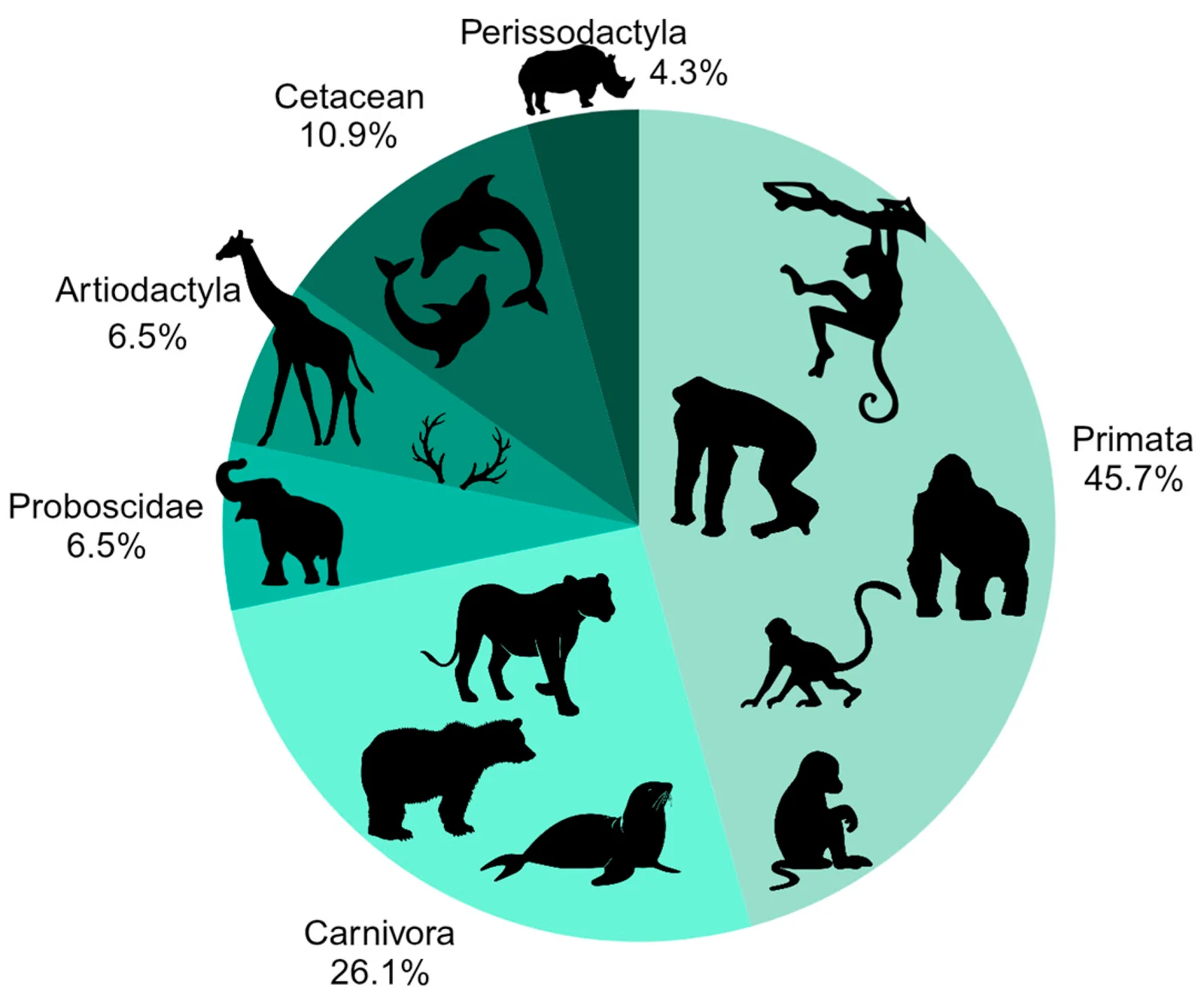
Percentage of studies analyzed according to the scientific classification of animal orders in the 46 published articles.

According to the International Union for Conservation of Nature (IUCN) [24], there are currently 6446 mammal species listed, classified into 27 different orders. However, this review identified studies only for six orders, focusing on primates, carnivores, proboscideans, artiodactyls, cetaceans, and perissodactyls. Figure 2 illustrates the proportion of species examined within each order, highlighting the representativeness of each group in the context of this review.

**Figure 3:**
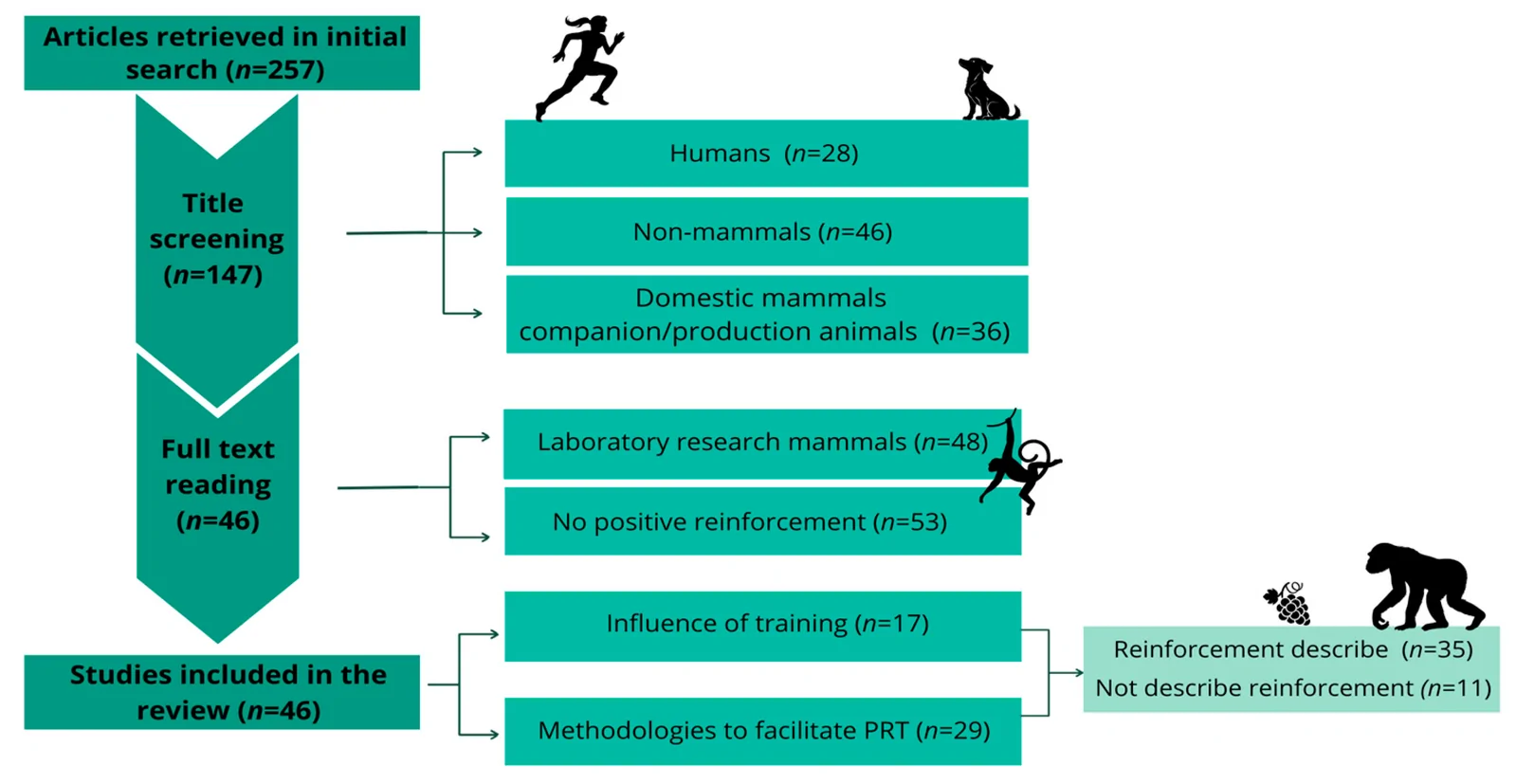
Number of publications included and excluded, published between 2014 and August 2024, regarding Positive Reinforcement Training (PRT).

The database search yielded a total of 257 articles. Following screening and eligibility assessment, 46 articles were selected for inclusion in this review. Among these, 17 focused on analyzing the influence of PRT on animal physiology, behavior, and welfare. The remaining 29 articles investigated husbandry and treatment strategies that employed training as a methodology to facilitate experimental procedures. In 35 of the reviewed articles, the types of reinforcers used were explicitly described (Figure 3). The following Table 1 summarizes the selected studies, emphasizing their primary focus and training objectives.

## 4. Effect of Positive Reinforcement Training on the Quality of Life of Animals

In this section, we examine the impact of PRT on animals’ quality of life, assessed through physiological indicators and analysis of their emotional, behavioral, and physiological states [1]. Operant conditioning with positive reinforcement reduces negative states in animals [60], fostering greater confidence in the presence of unfamiliar observers [54,59]. This approach not only enhances animals’ willingness to interact with humans but also increases their receptivity to social interactions.

However, introducing new, invasive commands—even for animals familiar with the technique—may not yield immediate success. For example, in chimpanzees (*Pan troglodytes*), training for blood glucose testing highlighted the importance of factors such as sex, receptivity, and prior anesthetic experiences, emphasizing the necessity of tailoring methods to individual characteristics to optimize outcomes [34].

In an ex situ conservation context, consistent conditioning can positively influence reproductive success. The more days dedicated to training within a month, the higher the likelihood of successful reproductive endeavors [40]. Recent studies have used saliva [28,42,44,46,49,55], blood [47], and fecal samples [48] to analyze stress-related hormone levels. Techniques such as infrared thermography are employed to measure changes in nasal temperature associated with emotional states [42], while behavioral responses to various situations—stressful or not—are also studied [44,48,49,54,59] (Table 2).

### 4.1. Behavioral Analysis

Behavior is understood as a set of actions and reactions of animals in response to internal physiological stimuli and external environmental factors [61], functioning as an indicator of health and well-being [9]. Behavioral analysis goes beyond merely observing animals’ actions; it involves applying scientific methods to identify needs, understand behaviors and foster positive interactions [10].

The literature indicates that a species’ behavioral repertoire is influenced by diverse factors, such as the trainer [62], biology, population characteristics, and individual traits—all of which are relevant for adaptation and reproductive success [61]. Animal individuality influences learning processes, such as in positive reinforcement training (PRT), through temperament and social dynamics [34]. Accordingly, inter-individual variation affects training efficacy and the number of sessions required [54,59].

In this context, PRT has proven to be a tool for improving animal welfare. For example, in jaguars [49] a negative correlation exists between stress levels and activity, while one increases the other decreases. Similarly, in ring-tailed lemurs [54], an increase in inactivity following training is considered positive because it reflects engagement during sessions. Furthermore, participation in PRT is voluntary [10], which indicates that animals often perceive it as beneficial, enhancing their psychological well-being and reducing stress.

Moreover, PRT is not only an instrumental behavioral but also serves as a mechanism for early disease detection. In dolphins, for instance, the willingness to participate in training correlates positively with signs of good health and higher food intake [38]. Regular and predictable training enriches animals’ behavioral repertoire, improves environmental engagement and supports emotional health [36,44,63].

A significant gap in the current literature is the limited exploration of the influence that trainers have on training outcomes. Many studies overlook the contingency between request, response, and reinforcement, and often do not thoroughly examine the communication preferences of the animals. Given that animals have diverse sensorial perceptions and preferences, understanding these differences is noteworthy—not only because they directly impact learning and training effectiveness but also because respecting individual preferences fosters greater trust and a stronger relationship between animals and trainers.

For example, during training, social isolation of social animals such as ring-tailed lemurs (*Lemur catta*) [54], vervet monkey (*Chlorocebus aethiops*) [59], and bottlenose dolphins (*Tursiops truncatus*) [30] can lead to increased stress-related behaviors, including lip licking and excessive vocalizations. Conversely, social reintegration has been shown to reduce agonistic behaviors and increase positive social interactions [54,59], as well as improve communication vocalizations [30].

The acoustic characteristics and duration of the speech used by trainers should be considered, as they can influence animal responses during sessions. Speaking in a pleasant tone has been associated with increased approach behavior and greater willingness to engage, whereas reprimanding tones can cause withdrawal and reduced response accuracy [46]. Respecting animals’ communication preferences, as observed in Bornean orangutans (*Pongo pygmaeus*) [29], where gestures are preferred over words, gazes, or combined signals, facilitates trust-building and enhances training success. Prioritizing gesture-based communication encourages greater engagement and procedural success.

Similarly to humans, nonverbal communication—including body language, facial expressions and other cues—plays a role in the learning process. This form of interaction influences attention, understanding, and motivation [64], providing animals with information about human intentions, emotional states, and responses [65,66]. However, this aspect remains underexplored in current research, leaving a gap in our understanding of how animals’ communication preferences might optimize training outcomes.

Although the benefits of PRT in reducing anxiety, stereotypic behaviors [44] and promoting positive interaction [54], are well established, there is still a considerable methodological gap across studies. For instance, in a review of 46 articles on animal training, only 12 (approximately 26%) mentioned the use of video recordings to document training sessions. This is concerning because video-based documentation ensures precise, objective, and replicable data collection. It allows for detailed analysis of subtle behaviors, facial expressions, and physiological responses (including the occurrence of feces, urine) that might be missed in real-time observation Additionally, videos enable thorough review, validation of results, identification of behavioral patterns over time, and accurate monitoring of individual progress. Incorporating systematic video analysis is indispensable for strengthening the scientific rigor of behavioral research and, ultimately, advancing animal welfare practices.

### 4.2. Cortisol Analysis

Among the 17 studies that focused on analyzing the influence of PRT on animal physiology and behavior, only 8 (approximately 47%) used glucocorticoid analysis. Exposure to mild to moderate stressors of short duration can be adaptive and beneficial for animals. Cortisol analysis was employed to assess the impact of PRT on animals, enabling comparisons of cortisol levels before, during, and after training sessions, as well as in stressful situations such as sedation or exposure to environmental enrichment (EE) items. Cortisol, commonly known as the “stress hormone,” tends to increase in response to activation of the hypothalamic–pituitary–adrenal (HPA) axis, which occurs during disruptions of homeostasis [67]. Animals under human care should not be entirely shielded from stress, as stressors related to activities such as hunting and reproduction are part of their natural survival process in the wild (in situ) [10]. Therefore, management practices can focus on optimizing stress levels to promote healthy physiological and behavioral functioning, rather than eliminating.

Analyzing variations in salivary cortisol concentrations across different species during veterinary management and PRT reveals distinct response patterns. In bonobos *(Pan paniscus*) and Sumatran orangutans (*Pongo abelii*), no significant changes in salivary cortisol levels were observed, indicating that PRT did not markedly influence their stress responses [28]. Conversely, in jaguars (*Panthera onca*), cortisol levels appeared to increase during conditioning, followed by a decrease, suggesting a more intense response to training [49]. Similarly, in grey wolves (*Canis lupus*), cortisol concentrations decreased from pre- to post-training [46], and in gorillas (*Gorilla gorilla*), reductions were reported following conditioning sessions [42,44]. However, given the small sample sizes in the studies using cortisol as a physiological indicator, it is difficult to draw definitive conclusions about the effects of PRT on stress responses based solely on these reported values.

A study using salivary analysis was conducted with African elephants (*Loxodonta africana*), comparing cortisol levels during PRT and exposure to a novel object (EE) [55]. Results indicated that PRT was associated with a relative decrease in cortisol levels, consistent with findings in gorillas [44] and wolves [46], whereas exposure to EE elicited an increase [55]. However, this elevation may not reflect negative stress; it could represent excitement or anticipation, such as eagerness to engage in play. For example, in grey wolves, individual preferences for stimuli were evenly split, with 50% choosing EE stimuli and 50% favoring training stimuli [11]. This variation underscores the complexity of behavioral responses and emphasizes the importance of tailoring strategies to individual animals’ needs. It is also recommended the inclusion of multiple measures of animal welfare in each study to account for the complexity of the behavioral aspects involved.

Similarly to the salivary cortisol analyses in bonobos and Sumatran orangutans, where no significant changes were observed [28], the assessment of fecal glucocorticoid metabolites (FGMs) in Indian rhinoceroses (*Rhinoceros unicornis*) also showed no notable variations. These findings suggest that operant conditioning with positive reinforcement was not perceived as a stressor by the species studied in the referenced studies [28]. However, given the small sample sizes typical of this investigation, such interpreted with caution. Factors such as prior experiences [47,48], age, sex, and interactions with humans [28] can influence hormonal responses and may modulate stress levels.

These results show that PRT can reduce stress during veterinary procedures in wild animals, especially when compared to chemical restraint. For instance, cortisol levels in trained grizzly bears (*Ursus arctos horribilis*), which remained below the detection limit (<1.7 ng/mL), were markedly lower than those of chemically immobilized bears—whose levels averaged 80.2 ± 63.1 ng/mL, indicating that operant conditioning effectively minimizes physiological stress and promotes calmer behaviors. While immobilized bears often exhibited negative reactions such agitation and vomiting at the sight of sedation equipment, trained bears remained calm during blood collection, voluntarily presenting limbs and allowing procedures to be performed without distress [47]. This approach significantly enhances animal welfare by enabling more humane and less invasive management. It is worth emphasizing that blood sampling protocols included two different formats: in one, animals were trained to voluntarily present limbs and in the other, they were sedated as a control. These findings underscore the value of training that can reduce stress and foster voluntary cooperation.

These findings regarding salivary cortisol and FGM across different studies reveal a complex picture. While some studies suggest that PRT may be linked to stable or lower glucocorticoid levels, implying a possible reduction in stress, it is noteworthy to acknowledge that cortisol levels are not a simple or definitive indicator of stress or overall well-being. Cortisol is involved in a range of physiological processes, including metabolic regulation and energy mobilization, and its levels can fluctuate due to factors such as arousal, anticipation, or individual differences in response to stimuli. Consequently, cortisol measurements should be interpreted cautiously and within the broader context of an animals’ behavioral and physiological state. The conflicting results reported in the literature showing decreases, others no change and some increases in cortisol levels following PRT, highlight this complexity. Therefore, cortisol should be considered as one element within a multifaceted approach to assessing animal welfare rather than a definitive indicator. To better understand the impact of PRT on animal welfare, glucocorticoid data should be combined with behavioral observations, other physiological measures, and the environmental context.

Details about the environment, including the training location, presence of distractions, and the use of any cues or signals, should be clearly documented. The documentation would enhance the practical application of the findings and support the progress of animal conditioning research, ultimately resulting in more robust and reliable outcomes.

## 5. Applications of Positive Reinforcement Training in Scientific Research

**Figure 4:**
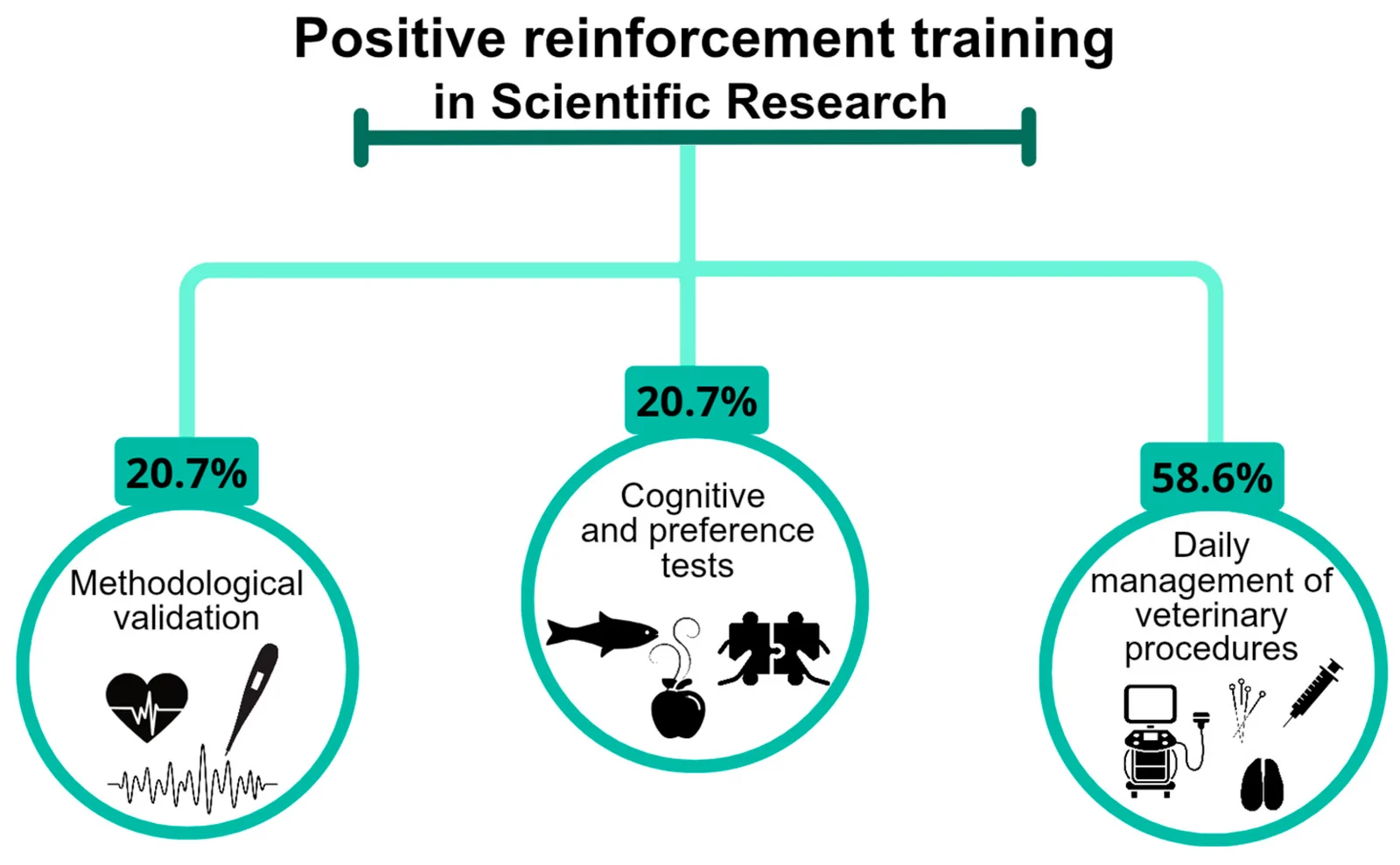
Percentage of studies on positive reinforcement training with focused groups organized by study objectives.

This section highlights how training, particularly PRT, is employed as a tool to facilitate the application of experimental procedures in research with diverse scientific objectives, where PRT was not necessarily the primary focus. PRT aids data collection by minimizing the effects of animal handling on endocrinological (hormonal) and behavioral parameters that could potentially confound results. The reviewed studies demonstrate that positive reinforcement techniques are effective in obtaining a wide range of biological and behavioral data, underscoring their utility in ex situ contexts. These studies have been categorized based on their objectives into three groups, as illustrated in Figure 4.

Additionally, the details of the 29 references are summarized in Table 3.

### 5.1. Training Focused on Validating Data Collection Methodologies

Positive reinforcement training has been effectively employed to validate data collection methodologies in studies involving wild animals, particularly in ex situ environments. Out of 29 reviewed articles, only 6 addressed validations of methods through training.

For example, in a study involving slow loris (*Nycticebus bengalensis*), the use of accelerometers attached to collars was voluntarily accepted by the animals, allowing for precise monitoring of activity patterns for ecological and behavioral research [19]. Similarly, chimpanzees (*Pan troglodytes*) were conditioned to accept electrodes on their chests, while bonobos (*Pan paniscus*) were trained to hold sensors [27], enabling the collection of heartbeat data without the need for anesthesia. In the case of Chinese water deer (*Hydropotes inermis*), positive reinforcement facilitated the insertion of thermometers for temperature measurement, aiding in the establishment of normal physiological ranges [35].

In microbiology research, PRT has enabled the voluntary collection of oral samples from golden monkeys (*Rhinopithecus roxellana*), facilitating detailed microbiota analysis. This method could identify pathogenic bacteria and assess oral health [41]. Additionally, conditioning with positive reinforcement proves to be a powerful tool for exploring vocal creativity and complex vocal learning in a voluntary and satisfactory manner, especially in studies involving savannah elephants (*Loxodonta africana)* [56].

PRT offers more reliable results and [32] is less stressful than traditional methods such as chemical restraint [68]. For instance, in a study involving grizzly bears, sedation was shown to induce significant physiological and behavioral changes that could compromise data integrity and methodology validation [47]. However, there are still few studies that have validated measurement methods or analyses, and the range of species studied remains limited.

Research conducted in zoos, aquariums, conservation centers and research facilities face unique challenges, such as a limited sample number of individuals of the same species in these institutions. Additionally, geographical distances hinder extensive studies across multiple locations. These limitations are compounded by individual differences, such as age, sex and social hierarchy, which can influence research outcomes. Despite these challenges, PRT improves the intervention quality. Its expansion and adaptation across diverse species are necessary to increase scientific robustness while promoting animal welfare. An important strategy to enhance data reliability and validity involves comparing behaviors, mental states, and physiological responses between trained and untrained animals, or assessing these variables in the same individuals before and after training. Furthermore, if these environments accurately quantify training data and integrate it into ZIMS (Zoological Information Management System)—a global platform for managing and sharing information on species and husbandry—we would have a valuable resource capable of transforming research and animal management into more efficient, reliable, and integrated tools.

### 5.2. Training Focused on Cognitive and Preference Tests and the Range of Species Studied Remains Limited

PRT has been employed to conduct preference tests and cognitive assessments, fostering the development skills across various species, such as grey seals (*Halichoerus grypus*) [45], bottlenose dolphins [37], mouse lemurs (*Microcebus lehilahytsara* and *M. murinus*) [51], spider monkeys (*Ateles geoffroyi*) [57], killer whale (*Orcinus orca*) [50], and American black bears (*Ursus americanus*) [25]. This approach relies on associative learning, whereby specific reinforcers create a positive environment that facilitates problem-solving and the execution of complex tasks.

Research has demonstrated that PRT effectively increases dolphins’ interaction with EE items and objects they may have previously ignored [69] or toward which they previously exhibited fear [37]. By stimulating activity, boosting energy expenditure, and diversifying behaviors, the method enhances aquatic mammals’ engagement with these stimuli [36], leading to improved learning outcomes. Consequently, conditioning helps these animals to accept new challenges and develop cognitive skills [50].

Furthermore, training plays a role in cognitive testing by enabling animals to learn and demonstrate their abilities. For instance, seals require an initial conditioning phase to develop skills for interacting with choice devices [37]. In studies with mouse lemurs (*Microcebus lehilahytsara* and *M. murinus*), it was observed that they can distinguish between conspecific and heterospecific urine odors [51]. Similarly, spider monkeys (*Ateles geoffroyi*) have successfully associated odors from ripe and unripe fruits [57]. These individual capabilities are vital for successful training, emphasizing the importance of adapting methods to the behavioral characteristics of each species. Different taxa exhibit varied responses to training procedures, highlighting the need for approaches tailored to specific behavioral and cognitive traits.

In studies involving specimens of the American black bear (*Ursus americanus*) [25] and orcas (*Orcinus orca*) [50], animals demonstrated effective learning in manipulating objects and selecting rewards based on specific criteria. These practices not only enhance their cognitive skills but also reduce anxiety related to uncertainty about rewards [45]. The findings illustrate that PRT can be effectively used in cognitive testing, enriching the experiences of bears [25], improving the welfare of killer whales [50], and providing valuable insights into seal foraging behaviors [45].

Despite the increasing use of positive reinforcement training as a tool to enhance the welfare and management of mammals under human care, there remains a significant gap in understanding their cognitive processes and food preferences. Recognizing individual differences, particularly regarding dietary preferences, is essential for developing more effective, species- and individual-specific training strategies. However, given the vast diversity of species and variation in cognitive capacities, further research would help to elucidate how different animals perceive, process information, learn, and select their food. A review of the literature indicates that, out of a total of 46 studies on the subject, only 6 (approximately 13%) addressed cognitive aspects or food preferences. This limited representation underscores the insufficiency of current knowledge to inform more personalized and efficient management practices.

### 5.3. Training Focused on Daily Management and Veterinary Procedures

Daily management and veterinary procedures training can facilitate routine activities and improve animal health care. It enables animals to voluntarily participate in procedures that can otherwise be invasive and stressful [10]. This approach reduces reliance on physical and chemical restraint methods, which pose significant risks to the animals’ safety [18]. Such methods can induce stress and negatively affect natural and reproductive behaviors [70], which is relevant in the context of species reintroduction and conservation efforts.

Through PRT, procedures that previously required invasive techniques can be performed voluntarily by the animals, leading to increased precision and reliability in physiological measurements across various species. A well-structured conditioning program, with clearly defined goals [10], combined with consistency and appropriate variation, is fundamental to maintaining animals’ focus and motivation throughout training. The caregivers act both as primary trainers and supporters, emphasizing the importance of tailoring strategies to each animal’s temperament to optimize outcomes [15,22,31,33,44].

In preventive medicine, PRT facilitates routine health checks, such as blood pressure monitoring, CT scans, and dental treatments, without the need for sedation [71].

Among primates, particularly *Strepsirrhini*, 97% of conservation organizations have reported improvements in human–animal interaction and psychological well-being resulting from the use of PRT [58]. Despite challenges such as slow progress and variable motivation, conditioning has demonstrated effectiveness in practices like the use of scales and cage acclimation. During the COVID-19 pandemic, this methodology proved invaluable in managing chimpanzees (*Pan troglodytes*), training them to move safely between stalls without direct human contact [12].

Structured group training has also proven effective in teaching complex, socially relevant behaviors, as seen in cheetahs (*Acinonyx jubatus*) [31] and mandrills (*Mandrillus sphinx*) [15]. Dividing animals into smaller subgroups enhances learning, maintains focus, and reduces stress and aggression related to resource competition [31]. This strategy fosters a cooperative and healthy environment, minimizing conflicts. Species exhibiting gregarious behavior in group settings should avoid excessive individualization in training, as social isolation can increase stress.

Breeding and raising offspring should be managed to minimize stress. In this regard, training females to use ultrasound for monitoring fetal development has proved effective for species such as Eastern black-and-white colobus monkey (*Colobus guereza*) [21] and Sumatran orangutan (*Pongo abelii*) [20]. This practice helps determine birth dates and allows early detection of complications, reducing stress associated with frequent veterinary interventions. Training mothers to voluntarily present their offspring for health checks, particularly when initiated during pregnancy, can further facilitate monitoring and reduce maternal defense behaviors [33] However, prior negative experiences with veterinary procedures may necessitate more training sessions to achieve success [20].

Studies have shown that positive reinforcement training facilitates veterinary procedures across multiple species, including deformed nail treatment in Siberian tigers (*Panthera tigris altaica*) [22], bathing in elephants for tuberculosis testing [39], and hoof care in giraffes [52,53] and elephants [55,60]. This approach makes treatments less invasive and offers numerous advantages. For example, voluntary collection of vaginal swabs and blood samples from lionesses has proven to be effective and minimally invasive, eliminating the need for frequent anesthesia [18].

In the case of black rhinoceroses (*Diceros bicornis*), operant conditioning facilitated blood collection via the transverse facial vein, a procedure for monitoring anticonvulsant levels in cases of idiopathic epilepsy [14]. Similar techniques have been successfully applied in spectacled bears and Asiatic black bears (*Ursus thibetanus*), demonstrating that blood sampling can be performed without specialized equipment, solely through positive reinforcement training [26].

Beyond routine care, PRT can be integrated into physiotherapy and rehabilitation programs, as well as in alternative treatments, and can be used to redirect undesirable behaviors such as self-injury. These interventions improve mobility and overall physical health [5,43]. For example, in procedures such as acupuncture [72], conditioning allows for electroacupuncture without anesthesia; this was successfully performed on a Bengal tiger (*Panthera tigris tigris*) with deforming spondylosis, which regained mobility [13]. Although PRT presents promising advantages for improving animal welfare, management practices and behavioral outcomes, its application must be critically evaluated. PRT should not be regarded as a universal solution or a risk-free intervention. In the context of reintroduction program, modifications to natural behaviors could interfere with survival skills. Without proper scientific assessment, the use of such techniques may undermine animals’ ability to adapt and survive effectively in their natural habitats. Additionally, the absence or inadequate documentation of training sessions through video recordings hampers the thorough evaluation of training effectiveness and animal responses. Therefore, the implementation of PRT should be conducted with caution, ensuring that evidence-based criteria are met for each specific case, to maximize benefits while minimizing potential risks to animal fitness and conservation success.

## 6. Type of Reinforcers

An analysis of several studies indicates that information regarding the types of positive reinforcement employed in training animals under human care is not consistently detailed in scientific publications. Out of the 46 articles reviewed, only 35 provided specific information on the reinforcers used. Table 4 presents the main types of primary and secondary reinforcers identified in these studies, along with their characteristics and applications.

These reinforcers help facilitate cooperation, as observed in chimpanzees [32] and savanna elephants (*Loxodonta africana*) [55], as well as promoting greater active participation in gorillas [42]. They also contribute to increased permanence in designated areas (such as on the scale or inside the stall), as observed with mandrills and lionesses [15,18], and support greater tolerance to handling black rhinos (*Diceros bicornis*) [14].

The effectiveness of positive reinforcement hinges on its capacity to enhance task performance during training. In this context, the term “positive” refers to the addition of elements that encourage animals, such as food, praise, or petting. The reinforcers must be tailored to each animal, as reward preferences vary. When working with multiple animals, observing individual preferences allows for adjustments in reinforcement strategies, which can improve training outcomes [52].

Food-based reinforcers typically involve items that are outside the animals’ usual diet [25,33], such as special treats or additional food not available in their daily diet. This approach aims to increase motivation by offering more attractive rewards. However, selecting these rewards requires careful consideration of constraints such as caloric intake and nutritional balance, which vary between species and individuals. For instance, a study involving *Loxodonta africana* and *Elephas maximus* found a correlation between high body condition scores (BCSs) and increased frequency of PRT sessions [73]. In such cases, it is vital to ensure that rewards are consistent with the animals’ regular diet to promote acceptance without jeopardizing health or well-being. These foods can be integrated into daily diet plans, with adjustments made to the overall nutritional regimen to maintain balance [5].

Out of the 35 articles describing the items of reinforcement used, only 10 (≈28.57%) reported the use of foods belonging to the animals’ regular diet, while 11 (≈31.43%) employed foods outside their usual diet. Additionally, 14 (about 40%) studies did not specify the reinforcement item used. None of the 25 studies that did not utilize foods from the animals’ standard diet addressed or quantified the caloric content of the reinforcers. This lack of detail makes it difficult to assess the nutritional impact and the raises concerns regarding the health risks for the animals.

The preferred foods of mammals, as reported in the reviewed studies [28,33,41,48,52], range from fruits in omnivores to meat in carnivores. Examples of primary reinforcers used in training include 2 to 3 cm chunks of beef for cheetahs [22,31], a mixture of honey and water (50:50) for grizzly bears [47], 3 cm pork cubes for Siberian tigers [22], beef pieces weighing between 50 and 75 g plus a chicken at the end for lionesses [18], and 1.5 cm gelatin cubes used with ring-tailed lemurs (*Lemur catta*) and vervet monkeys (*Chlorocebus aethiops*) [54,59].

The characteristics of rewards, such as size, shape, and amount, can vary widely and are influenced by factors like the session, the individual animal, the species, and the specific protocols of the institution. As such, successful training relies on the appropriate use of primary and secondary reinforcers—delivered in the right quantity, at the right moment, and in consideration of the animal’s particular needs. While careful adaptation to each context and avoiding frustration are relevant, these variables should be systematically adjusted and thoroughly documented to ensure transparency in methods and results.

Furthermore, secondary reinforcers—such as auditory stimuli (clickers and whistles) and social cues like praise and affection—are commonly used to enhance training effectiveness. Most studies indicate the use of continuous reinforcement, where animals are rewarded immediately after exhibiting the correct behavior [28,50]. However, some approaches, such as those employed with gorillas [42] and lionesses [18], use *variable-ratio reinforcement method*, in which rewards are delivered at unpredictable and varying intervals. While classical learning theory demonstrates that variable ratio schedules can produce very strong behaviors that are highly resistant to extinction (e.g., analogous to behaviors observed in human gambling), their application in positive reinforcement training aimed at animal welfare and promoting cooperation with captive wild mammals requires nuanced considerations. The inherent uncertainty regarding the timing of reward, although it can promote engagement in certain scenarios, may also increase anxiety and frustration in some individuals. This could shift the animals’ focus from the commands to anticipating the reward, potentially hampering the development of clear and consistent associations between behavior and consequence—an essential aspect for training efficacy and trust-building. Additionally, inconsistent application of primary reinforcers may, over time, influence the animals’ perception of control, thereby diminishing the effectiveness of secondary reinforcers used as feedback mediators, leading to reduced motivation and attention, especially when the goal is voluntary, stress-free participation in management procedures.

The selection of reinforcers plays a role in the success of operant conditioning. Just as primary reinforcers should be chosen based on the animal’s preferences, physical condition, health, and training goals, secondary reinforcers also need to be selected with consideration of how the animals perceive them, since many species do not see or hear in the same way humans do. Variations in perception and acceptance of this stimulus across different species and individuals can influence their effectiveness in encouraging desired behaviors. Using both primary and secondary reinforcers thoughtfully can help improve training outcomes, motivation and overall animal welfare.

## 7. Final Considerations

Guiding positive reinforcement training based on principles that promote animals’ well-being, safety, and effectiveness is necessary. While there is no single universal formula, it is advisable to adopt criteria such as detailed session planning, the application of specific techniques, such as differential reinforcement of incompatible behaviors and differential reinforcement of other behavior, along with establishing progress criteria. Equally, the systematic recording and monitoring of the animal’s responses. Information on structuring a training program can be found in the guidelines of EAZA [74], as well as in institutional reports from IMATA, WAZA, and ALPZA, which offer practical guidance for the application of these techniques.

Another aspect that is seldom addressed in the literature is the training of the team responsible for animal training. Proper qualifications of professionals can influence the success of this technique. Several institutions, such as EAZA and AZA, offer specific training courses available on their official websites [75,76]. Including information about staff training and standardization, as well as the impact of proper training on intervention outcomes, could significantly contribute to the advancement of management and training practice.

PRT encompasses a wide range of actions, including daily handling, veterinary procedures, collection of biological samples, and cognitive testing. In this context, guidance should prioritize flexible principles and strategies that can be adapted to diverse objectives and the unique characteristics of each species. Such principles, aligned with best management and welfare practices, serve as a foundational framework for designing and executing training programs. Moreover, sharing success or outcomes, challenges, and failures is vital for fostering the exchange of experiences. Transparency not only enhances the credibility of findings but also facilitates their practical application across various settings—from zoos to conservation centers.

A primary issue in current methodologies is the insufficient description of procedures used. Additionally, results that are statistically non-significant, inconclusive, negative, or others are often not published, remaining within gray literature. This absence of data can limit the understanding of the true research landscape and may bias conclusions, favoring a more optimistic or exaggerated view of the current state of knowledge. Therefore, publishing such results in peer-reviewed journals broadens the evidence base, promotes a more balanced analysis, and ensures a more scientifically grounded evaluation.

To strengthen this research area, future publications should provide detailed descriptions of the methodology, particularly regarding the characteristics of the reinforcement used, its nature, and the method of administration. Such detailed reporting would facilitate the evaluation and adaptation of strategies to individual differences. Moreover, integrating video recordings with AI technology represents a promising avenue for monitoring, quantifying behaviors, and providing real-time recommendations to optimize training protocols. These innovations can advance the rigor and effectiveness of behavioral assessments, paving the way for more personalized and scientifically robust animal management practices, future research should explore the full potential of these tools.

To optimize results, efforts can be invested in the personalization of training programs, considering the particularities of each animal, such as temperament, age, and social behavior. Differences in maternal care, for example, the behavior of deer and lions that leave their young to feed, in contrast to elephants and rhinos that keep their young close, can influence performance during training. By considering behavioral and physiological variables, designing training programs more aligned with the characteristics of each animal increases the chances of success.

Studies related to animal welfare, biological material collection, practical applications of conditioning can benefit from collaboration between researchers and institutions. Such partnerships promote cooperation in research projects, conservation advancements and educational initiatives. Institutions such as wildlife conservation and rehabilitation centers, zoos, and aquariums are recommended to publish articles detailing their training plans, including objectives, locations, schedules, strategies adopted, and types of rewards and fill out the ZIMS. Having detailed information is key to sharing best practices and fostering improvements in animal training routines. To allow progress to be tracked and generate reliable data for future publications, we need to keep field records of each session. Highlighting the principles and strategies adopted also contributes to the dissemination of effective references. Thus, this exchange of information enriches knowledge and favors the development of ethically aligned training.

## Figures and Tables

**Table 1:** Data from the 46 articles included in the review are organized as follows: (A) the effect of positive reinforcement training on the quality of life of animals; and (B) applications of positive reinforcement training in scientific research.

Popular Name (Scientific Name)	Sample Size	Training Study Focus	Primary/Secondary Reinforcement	Training Objective	Training Schedule	Training Duration	Reference
American black bear (*Ursus americanus*)	1	A	Yes	Cognitive test for discrimination	13:00 h	10 attempts	[25]
Bear (*Tremarctos ornatus and Ursus thibetanus*)	2 and 2	A	Yes	Blood collection	-	10–15 min	[26]
Bengal slow loris (*Nycticebus bengalensis*)	1	A	Yes	Allowed for the attachment of the accelerometer for validation with reduced stress for the animal	During the night	15 min	[19]
Bengal tiger (*Panthera tigris tigris*)	1	A	Yes	Allowed veterinarians to perform acupuncture safely without anesthetizing	-	16 volts of 10 min	[13]
Black rhinoceros (*Diceros bicornis*)	1	A	Yes	Desensitization to a needle puncture in the facial vein	-	20 min	[14]
Bonobo (*Pan paniscus*)	7	A	No	Obtain an ECG reading	-	-	[27]
Bonobos (*Pan paniscus*) e Sumatran orangutan (*Pongo abelii*)	10 and 7	B	Yes	Salivary cortisol levels in stress and baseline conditions	14:00 h ± 10 min	Exactly 20 min	[28]
Bornean orangutan (*Pongo pygmaeus*)	2	B	Yes	Influence of different training instruction methods	Winter 16:00 h–17:00 h Summer 17:00 h–18:00 h	90 commands	[29]
Bottlenose dolphin (*Tursiops truncatus*)	8	B	Yes	Vocal behavior during the interactions	±5 sessions per day	15 min	[30]
Cheetah (*Acinonyx jubatus*)	8	A	Yes	Develop a methodology for group training	After feeding	5 min	[31]
Chimpanzee (*Pan troglodytes*)	35	A	Yes	Obtain an ECG reading	During feeding	2 min	[32]
Chimpanzee (*Pan troglodytes*)	2	A	No	Assist the staff with daily management procedures	-	-	[12]
Chimpanzee (*Pan troglodytes*)	18	A	Yes	Implement and obtain an initial evaluation of a physical therapy program	-	4–5 min	[5]
Chimpanzee (*Pan troglodytes*)	3	A	Yes	Monitor the health status of babies and manage their feeding and treatment diseases	-	-	[33]
Chimpanzee (*Pan troglodytes*)	123	B	No	Blood glucose (BG) readings	-	5 min	[34]
Chinese water deer (*Hydropotes inermis*)	11	A	Yes	Rectal temperature measurement using a thermometer inserted into the rectum	14:30 h	5–10 min	[35]
Dolphin (*Tursiops truncatus* e *Tursiops aduncus*)	86	B	No	Comparative behavior	-	-	[36]
Dolphin (*Tursiops*)	4	A	Yes	Measured the effects of a PRT in the manipulation of objects	1–3 sessions per day	-	[37]
Dolphin (*Tursiops*)	51	B	Yes	Effect of training on animal welfare	5–10 sessions per day	-	[38]
Eastern black-and-white Colobus monkey (*Colobus guereza*)	3	A	Yes	Provide ultrasonographic fetal growth charts	-	-	[21]
Elephant	5	A	Yes	Proboscis washing (tuberculosis test)	7:30 h–10:00 h and 16:00 h–19:00 h	12 min	[39]
Fishing cat (*Prionailurus viverrinus*)	26	B	No	Influence on reproduction	-	-	[40]
Golden snub-nosed monkey (*Rhinopithecus roxellana*)	20	A	No	Collect 20 oral samples from three infants	-	-	[41]
Gorilla (*Gorilla gorilla*)	3	B	Yes	Access the influence and compare with other situations	7:00 h–8:00 h	30 min	[42]
Gorilla (*Gorilla gorilla*)	1	A	No	Physical examinations and application of physiotherapeutic exercises	-	-	[43]
Gorilla (*Gorilla gorilla*)	2	B	No	Evaluate salivary oxytocin and cortisol concentrations before and after PRT	±08:30 h	10–11 min	[44]
Grey seals (*Halichoerus grypus*)	5	A	No	Test prey species and size preferences	-	5–50 min	[45]
Grey wolf (*Canis lupus*)	9	B B	Yes	Salivary collection for cortisol analysis	8:30 h and 17:00 h	5 min	[46]
				Influence of intonation on the training process			
Grizzly bear (*Ursus arctos horribilis*)	8	B	Yes	Blood cortisol analysis: effect of training vs. sedation	22:00 h–23:00 h	5–10 min	[47]
Indian rhinoceros (*Rhinoceros unicornis*)	2	B	Yes	Observe the introduction of a conditioning program	7:00 h and 15:00 h	15–30 min	[48]
Jaguar (*Panthera onca*)	7	B	Yes	Investigate the effects of PRT	13:30 h–15:30 h	10 min	[49]
Killer whale (*Orcinus orca*)	1	A	Yes	Learn the rules of a delayed identity matching-to-sample task	1–4 sessions per day	12 attempts	[50]
Lioness (*Panthera Leo*)	6	A	Yes	Allow vaginal swab collection and blood withdrawal	-	5–10 min	[18]
Mandrill (*Mandrillus sphinx*)	4	A	Yes	Entrance to the food room	-	-	[15]
Mouse Lemur (*Microcebus lehilahytsara* e *M. murinus*)	4	A	Yes	Train animals to differentiate between the scent of conspecific and heterospecific urine	20 trials	Maximum 1 h	[51]
Reticulated Giraffe (*Giraffa camelopardalis reticulata*)	18	A	Yes	Obtain lateral and lateral oblique front foot radiographs	-	15 min and 1 h for collection days	[52]
Reticulated Giraffe (*Giraffa camelopardalis reticulata*)	1	A	Yes	Obtain dorsolateral palmar medial oblique front foot radiographs	7:00 h–8:00 h	15–20 min	[53]
Ring-tailed lemur (*Lemur catta*)	11	B	Yes	The effect on the behavior and welfare during temporary isolation PRT	1 time in the morning + 1 time in the afternoon	Up to 3 min	[54]
Savannah elephant (*Loxodonta africana*)	10	B	Yes	Salivary cortisol analysis: effect of training vs. environmental enrichment	13:00 h or 14:00 h or 18:00 h	10 min	[55]
Savannah elephant (*Loxodonta africana*)	5	B	Yes	Produce sounds by command	-	-	[56]
Siberian tiger (*Panthera tigris altaica*)	1	A	Yes	Collection, treatment and analysis	10:30 h and 16:30 h	10 min	[22]
Spider monkey (*Ateles geoffroyi*)	5	A	No	Perform the smell discrimination test	Morning	-	[57]
Strepsirrhini (*Primatas*)	71	A	Yes	Collect information about training program details, animals, behaviors, and techniques, the evaluation process, and the impact of training	-	5–10 min	[58]
Sumatran orangutan (*Pongo abelii*)	2	A	Yes	Ultrasound for pregnancy monitoring	-	-	[20]
Vervet monkey (*Chlorocebus aethiops*)	10	B	Yes	Could be trained to be isolated in a familiar area	-	Up to 15 min	[59]
Wolfs (*Canis lupus and Canis lupus arctos*)	4	B	Yes	Comparison of preference between EE and PRT	-	3 tests per day	[11]

**Table 2:** Summary of the 17 selected references, covering the species, specific objectives related to training, and the methods used to analyze the effect of training on the animals.

Popular Name (Scientific Name)	Objective of the Study	What Was Analyzed	Reference
Bonobos (*Pan paniscus*) e Sumatran orangutan (*Pongo abelii*)	Assess the effects of medical PRT on salivary cortisol in group training sessions	Salivary cortisol	[28]
Bornean orangutan (*Pongo pygmaeus*)	Understand which types of signals are more easily dealt with just words, just looks, just gestures, or all types of signals combined	Correct response to commands	[29]
Bottlenose dolphin (*Tursiops truncatus*)	Describe the possible effects of PRT on the emission rate of whistles	Whistles before, during and after the PRT	[30]
Chimpanzee (*Pan troglodytes*)	To assess which factors affect the initial training success of diabetic treatment training	Whether or not the test was performed	[34]
Dolphin (*Tursiops truncatus* and *Tursiops aduncus*)	Determine how EE, PRT, and habitat characteristics affect two potential indicators of animal welfare, repetitive behavior and behavioral diversity	Behavior	[36]
Dolphin (*Tursiops*)	Investigating four health and behavioral welfare measures	Health status, % of daily food eaten, occurrences of new rake marks, and willingness to participate (WtP)	[38]
Fishing cat (*Prionailurus viverrinus*)	Examine both the environmental factors and management practice	% of viable pregnancies	[40]
Gorilla (*Gorilla gorilla*)	Assess the effectiveness of infrared thermography (IRT) for measuring emotional responses to positive reinforcement training and cognitive tasks with familiar humans	Nasal temperatures associated with emotional change using IRT and saliva samples for hormone analysis	[42]
Gorilla (*Gorilla gorilla*)	Evaluated salivary oxytocin and cortisol concentrations before and after PRT	No change in OXT and CORT decreased in one subject following PRT	[44]
Grey wolf (*Canis lupus*)	Saliva was used for the physiological assessment of stress via measuring salivary cortisol	Did not find any effects on cortisol variation	[46]
Grey wolf (*Canis lupus*)	Explored how animals’ responses might differ in accordance with trainer’s communication style within a training session.	Friendly voice during training supports performance and positive emotional responses	[46]
Grizzly bear (*Ursus arctos horribilis*)	Described the evaluation of blood markers of physiological stress responses: Serum cortisol and immunoglobulin A (IgA) and plasma b-endorphin were measured as indicators of	Serum cortisol was undetectable in all trained bears, whereas chemically immobilized bears had marked cortisol elevations	[47]
Indian rhinoceros (*Rhinoceros unicornis*)	We sought to determine how management tools used for reproductive management, specifically translocation and operant conditioning, impact physiological and behavioral measures of welfare	Fecal glucocorticoid analysis and behavior	[48]
Jaguar (*Panthera onca*)	Aim to evaluate the effects of conditioning on the welfare of jaguars in captivity	Analyzing behavioral and physiological effects through and salivary cortisol	[49]
Ring-tailed lemur (*Lemur catta*)	Explore the role of an isolation PRT program on the well-being	PRT may play a crucial role for the captive management	[54]
Savannah elephant (*Loxodonta africana*)	Determine whether PRT and exposure to a novel object induced a salivary cortisol response	Salivary cortisol	[55]
Vervet monkey (*Chlorocebus aethiops*)	Evaluate whether a training program could be used to induce them to cooperate in behavioral management	The training program enriched the daily routine of these captive primates by increasing affiliative behaviors while decreasing agonistic behaviors	[59]
Wolfs (*Canis lupus* and *Canis lupus arctos*)	Test the efficacy of a preference assessment method using a trained behavior as a choice and test whether individuals prefer the performance of a trained behavior or an EE	50% of the subjects preferred trained behavior stimuli and 50% of the subjects preferred EE stimuli	[11]

**Table 3:** Summary of the 29 references analyzed, organized according to their research focus. For each study, the species investigated, and the specific training objectives are provided.

Training Focus	Popular Name (Scientific Name)	Objective of the Study	Results	Reference
Validating data collection methodologies	Bengal slow loris (*Nycticebus bengalensis*)	To investigate whether the PRT assists in the use of a collar equipped with an accelerometer and thus validate behavioral data	39 sessions were held and 10 behaviors linked to active and inactive were validated	[19]
	Bonobo (*Pan paniscus*)	Determine the feasibility and utility of the KM device for the determination of heart rate and rhythm	87 bpm	[27]
	Chimpanzee (*Pan troglodytes*)	Monitoring heart rate for reference points	Adult males = 86.5 beats per minute and females = 106.4 beats per minute	[32]
	Chinese water deer (*Hydropotes inermis*)	Measure body temperature, find the normal range	Average 39 °C	[35]
	Golden snub-nosed monkey (*Rhinopithecus roxellana*)	Performed sequencing in conjunction with bacterial isolation and identification to investigate age-related variation in the oral flora community composition	The oral cavity were Firmicutes and Proteobacteria (totaling 77.62%)	[41]
	Savannah elephant (*Loxodonta africana*)	Show variation in sound production of similar sounds by individuals	Identified high-frequency sound (HFS), the throb sound and the oral burst	[56]
Cognitive and preference tests	American black bear (*Ursus americanus*)	The use of judgment bias tasks to identify affect changes, focusing by seasonal variations in mulberry availability	He reached the criterion in just nine sessions (90 trials) and in session 6 he reached a level of 80% correct responses	[25]
	Dolphin (*Tursiops*)	Document the effect of the training program to stimulate the interest in enrichment objects in cases when animals show initial aversion or no interest	Object manipulation increased significantly	[37]
	Grey seals (*Halichoerus grypus*)	Investigate prey choice	Exhibiting size-based preferences and insights into the foraging behavior	[45]
	Killer whale (*Orcinus orca*)	Aimed to develop a perform a matching-to-sample task	Method was confirmed to be a useful tool for examining the visual abilities	[50]
	Mouse Lemur (*Microcebus lehilahytsara* and *M. murinus*)	Investigate the sensory olfactory capabilities in the context of species discrimination	Discriminated conspecific from heterospecific urine odor	[51]
	Spider monkey (*Ateles geoffroyi*)	Assess whether the odor profiles of fruits of these two species indeed have the potential to mediate the interaction between plants and primates	Able to discriminate the odor of ripe fruits from odors that simulate unripe fruits	[57]
Management and veterinary procedures	Bear (*Tremarctos ornatus* and *Ursus thibetanus*)	To develop a new voluntary blood collection method, allowing access to various veins	Facilitated blood collection and identified the cephalic vein	[26]
	Bengal tiger (*Panthera tigris tigris*)	A case that used traditional acupuncture techniques combined with animal training was a safe and effective alternative	Reacted positively after electroacupuncture was performed	[13]
	Black rhinoceros (*Diceros bicornis*)	Developed a technique to harvest facial veins without sedation	Blood was drawn from the transverse facial vein to monitor therapeutic levels of anticonvulsants	[14]
	Cheetah (*Acinonyx jubatus*)	Provide reference information	Ideal to train 2 animals at a time	[31]
	Chimpanzee (*Pan troglodytes*)	Implementation of training for care	Shifting animals calmly and voluntarily	[12]
	Chimpanzee (*Pan troglodytes*)	Create, implement, and evaluate the effects of participation in a physical therapy (PT) program	Improved physical health, increased mobility, increased activity levels, as well as greater well-being and a reduction in depressive behaviors.	[5]
	Chimpanzee (*Pan troglodytes*)	Training methodology for monitoring the health of juveniles	It began with pregnant mothers, and after giving birth, they voluntarily presented their offspring for care	[33]
	Eastern black-and-white Colobus monkey (*Colobus guereza*)	Provides gestational data	Established reliable fetal growth curves	[21]
	Elephant	Determine the effectiveness of secondary positive reinforcement in the voluntary participation in a trunk wash	35 sessions to have 89% success in collecting fluid for analysis	[39]
	Gorilla (*Gorilla gorilla*)	Promote and increase the understanding of conspicuous behavior as a sign of a malfunctioning and/or diseased organ	Redirect attention away from self-harming behaviors and simultaneously teach the animal to adopt positions necessary for physical assessments	[43]
	Lioness (*Panthera Leo*)	Assess the potential of PRT to voluntary access for the collection of vaginal swabs and blood samples	Collected of about 750 vaginal swabs and 650 blood samples over 18 months	[18]
	Mandrill (*Mandrillus sphinx*)	Training methodology for entering the food service area	Dominant animals allowed submissive animals to eat in peace.	[15]
	Reticulated Giraffe (*Giraffa camelopardalis reticulata*)	Describes the training process and restricted contact set-up for handling the front feet	Described the commands performed to x-ray	[52]
	Reticulated Giraffe (*Giraffa camelopardalis reticulata*)	Training methodology for paw radiography	Radiographs obtained within a 3-month period.	[53]
	Siberian tiger (*Panthera tigris altaica*)	Describe training methodologies	Implemented an entire training program to avoid sedation	[22]
	Strepsirrhini (*Primatas*)	We assessed the development and status of training programs with these species in North American institutions	Increased positive human–animal interactions and improvements in animals’ psychological well-being	[58]
	Sumatran orangutan (*Pongo abelii*)	Describe a case: with investigations and treatment	Allowed monitoring of pregnancy	[20]

**Table 4:** Presentation of the animals and the primary and secondary reinforcers used in training. A “-” indicates that the reinforcer was not specified in the article.

Popular Name (Scientific Name)	Primary Reinforcement	Component of the Basic Diet	Secondary Reinforcement	Reference
American black bear (*Ursus americanus*)	Almonds or dried cherries	No	Sound	[25]
Bengal slow loris (*Nycticebus bengalensis*)	Insect	-	Term “good”	[19]
Bengal tiger (*Panthera tigris tigris*)	Cattle beef	Yes	-	[13]
Black rhinoceros (*Diceros bicornis*)	Various foods or alfalfa cubes	-	-	[14]
Bonobos (*Pan paniscus*) e Sumatran orangutan (*Pongo abelii*)	Pieces of fruit	Yes	Clicker	[28]
Bornean orangutan (*Pongo pygmaeus*)	Piece of apple	-	Clicker and verbal praise	[29]
Bottlenose dolphin (*Tursiops truncatus*)	Fish	-	-	[30]
Cheetah (*Acinonyx jubatus*)	Cattle beef	No	-	[31]
Chimpanzee (*Pan troglodytes*)	Pieces of fruit	-	-	[32]
Chimpanzee (*Pan troglodytes*)	Grapes and juices	No and yes	-	[5]
Chimpanzee (*Pan troglodytes*)	Kiwi, juice, yogurt etc.	No	Clicker	[33]
Chinese water deer (*Hydropotes inermis*)	Carrots, grass and corn pallets	-	Clicker and whistle	[35]
Dolphin (*Tursiops*)	Food	-	Affectionate expressions, or ice cubes and whistles	[37]
Dolphin (*Tursiops*)	Fish and squid species	No	Rubs, attention, toys	[38]
Elephant	Chopped banana	No	Whistle	[39]
Gorilla (*Gorilla gorilla*)	Food	Yes	Clicker	[42]
Gray wolf (*Canis lupus*)	Pieces of gouda cheese	No	-	[46]
Grizzly bear (*Ursus arctos horribilis*)	Honey–water mixture	No	Clicker	[47]
Indian rhinoceros (*Rhinoceros unicornis*)	Peanut butter, apples, sweet potatoes, and alfalfa cubes	Yes	-	[48]
Jaguar (*Panthera onca*)	Cattle beef	No	Clicker	[49]
Killer whale (*Orcinus orca*)	Fish	-	Whistle	[50]
Lioness (*Panthera Leo*)	Red meat and chicken	Yes	Click and voice reward (“good!”)	[18]
Mandrill (*Mandrillus sphinx*)	Peanuts, raisins, dried dates and banana, egg and peach	Yes	Clicker	[15]
Mantled guereza (*Colobus guereza*)	Potato	Yes	-	[21]
Mouse Lemur (*Microcebus lehilahytsara* and *M. murinus*)	Apple juice and banana	-	-	[51]
Reticulated Giraffe (*Giraffa camelopardalis reticulata*)	Romaine lettuce or rye wafer crackers, but sometimes bananas, bread, or browse	No	Clicker	[52]
Reticulated Giraffe (*Giraffa camelopardalis reticulata*)	Fresh browse or romaine lettuce	Yes	Clicker	[53]
Ring-tailed lemur (*Lemur catta*)	Piece of primate jelly consisting of vegetables, fruits, nuts and honey (Viten^®^, Udine, Italy)	-	-	[54]
Savannah elephant (*Loxodonta africana*)	Bread	No	Clicker	[55]
Savannah elephant (*Loxodonta africana*)	Food	-	Verbal praise, patting and clicker	[56]
Siberian tiger (*Panthera tigris altaica*)	Pork beef	-	Clicker	[22]
Sumatran orangutan (*Pongo abelii*)	Grape	-	-	[20]
Ursos (*Tremarctos ornatus* e *Ursus thibetanus*)	Bear pellets (KS, Oriental Yeast Co., Ltd., Tokyo, Japan)	Yes	-	[26]
Vervet monkey (*Chlorocebus aethiops*)	Cube of jelly for primates consisting of a mixture of vegetables, fruits and nuts (‘Delicacy Gelée’ supplied by Viten^®^, Udine, Italy)	-	-	[59]
Wolfs (*Canis lupus* and *Canis lupus arctos*)	Natural Balance	Yes	-	[11]

## Data Availability

No new data were created or analyzed in this study. Data sharing is not applicable to this article.
